# IP-Se-06, a Selenylated Imidazo[1,2-*a*]pyridine, Modulates Intracellular Redox State and Causes Akt/mTOR/HIF-1*α* and MAPK Signaling Inhibition, Promoting Antiproliferative Effect and Apoptosis in Glioblastoma Cells

**DOI:** 10.1155/2022/3710449

**Published:** 2022-03-22

**Authors:** Daniela C. dos Santos, Jamal Rafique, Sumbal Saba, Valdelúcia M. A. S. Grinevicius, Danilo W. Filho, Ariane Zamoner, Antonio L. Braga, Rozangela C. Pedrosa, Fabiana Ourique

**Affiliations:** ^1^Laboratório de Bioquímica Experimental (LABIOEX), Departamento de Bioquímica, Universidade Federal de Santa Catarina (UFSC), Florianópolis, SC, Brazil; ^2^Instituto de Química (INQUI), Universidade Federal do Mato Grosso do Sul (UFMS), 79074-460 Campo Grande, MS, Brazil; ^3^Instituto de Química (IQ), Universidade Federal de Goiás-UFG, 74690-900 Goiânia, GO, Brazil; ^4^Departamento de Ecologia e Zoologia, Universidade Federal de Santa Catarina (UFSC), Florianópolis, SC, Brazil; ^5^Laboratório de Bioquímica e Sinalização Celular (LaBioSignal), Departamento de Bioquímica, Universidade Federal de Santa Catarina (UFSC), Florianópolis, SC, Brazil; ^6^Laboratório de Síntese de Substâncias de Selênio Bioativas (LabSelen), Departamento de Química, Universidade Federal de Santa Catarina, Florianópolis, 88040-900 SC, Brazil; ^7^Universidade Federal de Juiz de Fora, Departamento de Bioquímica, Centro de Ciências Biológicas, Juiz de Fora, MG, Brazil

## Abstract

Glioblastoma multiforme (GBM) is a notably lethal brain tumor associated with high proliferation rate and therapeutic resistance, while currently effective treatment options are still lacking. Imidazo[1,2-*a*]pyridine derivatives and organoselenium compounds are largely used in medicinal chemistry and drug development. This study is aimed at further investigating the antitumor mechanism of IP-Se-06 (3-((2-methoxyphenyl)selanyl)-7-methyl-2-phenylimidazol[1,2-*a*]pyridine), a selenylated imidazo[1,2-*a*]pyridine derivative in glioblastoma cells. IP-Se-06 exhibited high cytotoxicity against A172 cells (IC_50_ = 1.8 *μ*M) and selectivity for this glioblastoma cell. The IP-Se-06 compound has pharmacological properties verified in its ADMET profile, especially related to blood-brain barrier (BBB) permeability. At low concentration (1 *μ*M), IP-Se-06 induced intracellular redox state modulation with depletion of TrxR and GSH levels as well as inhibition of NRF2 protein. IP-Se-06 also decreased mitochondrial membrane potential, induced cytochrome *c* release, and chromatin condensation. Furthermore, IP-Se-06 induced apoptosis by decreasing levels of Bcl-xL while increasing levels of *γ*-H2AX and p53 proteins. Treatment with IP-Se-06 induced cell cycle arrest and showed antiproliferative effect by inhibition of Akt/mTOR/HIF-1*α* and ERK 1/2 signaling pathways. In addition, IP-Se-06 displayed significant inhibition of p38 MAPK and p-p38, leading to inhibition of inflammasome complex proteins (NLRP3 and caspase-1) in glioblastoma cells. These collective findings demonstrated that IP-Se-06 is a bioactive molecule that can be considered a candidate for the development of a novel drug for glioblastoma treatment.

## 1. Introduction

Glioblastoma multiforme (GBM) is the most common, invasive, and aggressive malignant primary brain tumor in adults [1]. It is associated with a poor prognosis, high recurrence, and high mortality rates, with median patient survival around 14.6 months after resection followed by adjuvant radiotherapy plus 6-12 cycles of temozolomide (TMZ) [2]. TMZ, a second-generation imidazotetrazine lipophilic prodrug, is a first-line chemotherapeutic drug for newly diagnosed glioblastoma [3]. However, TMZ benefits are limited by acquired chemoresistance, which represents a serious issue for glioblastoma therapy [4]. In addition, the poor permeability of anticancer drugs through the blood-brain barrier (BBB) can have important implications for drug delivery and efficacy [5]. Therefore, the development of a new and effective chemotherapeutic drug for the treatment of human glioblastoma is urgently required.

The glioblastoma microenvironment and alterations in signaling pathway proteins are pivotal to tumor progression and therapeutic resistance [1]. Recently, it has been reported that the PI3K/Akt/mTOR/HIF-1*α* pathway is involved in enhancing the migration and invasion of human glioblastoma cells under hypoxia [6]. Moreover, the activation of Akt/mTOR pathway is related to the development of drug resistance and decreased therapeutic effect of TMZ [2]. Rodríguez-García and collaborators [7] provided evidence of 6-phosphofructo-2-kinase/fructose-2,6-bisphosphate 3 (PFKFB3) involvement in glycolysis upregulation, in response to TGF-*β*1 in glioblastoma cells, which is mediated by Smad, p38 MAPK, and PI3K/Akt signaling. Furthermore, the MAPK/ERK signaling pathway is associated with tumor therapy resistance and invasive growth of glioblastoma [8]. Some studies have also emphasized the importance of the tumor microenvironment, particularly their inflammatory mediators and inflammasome activation for regulation of tumor development and progression [9].

Compounds containing the imidazo[1,2-*a*]pyridines (IPs) heterocycle system have been largely used in medicinal chemistry and drug development [10]. These compounds possess a wide spectrum of biological activity and pharmacological properties by binding on different targets [11]. Therefore, IPs have attracted critical attention due to the interesting properties exhibited in different types of molecular mechanisms in cancer chemotherapy [12] [13]. These compounds proved to be potent PI3K/mTOR dual inhibitors with excellent kinase selectivity [14], inducing cell cycle arrest and apoptosis [15]. In this regard, Quattrini and collaborators [16] reported the evidence of IPs derivatives as novel drug candidates for the treatment of glioblastoma.

On the other hand, selenium provides essential biological importance. Synthetic organoselenium compounds modulate a wide spectrum of biological processes, such as oxidative stress by thiol depletion, reactive oxygen species (ROS) overproduction, mitochondrial dysfunction, and DNA damage [17] [18]. The pharmacological activities of organoselenium compounds have been widely reported on, including their antitumor effects, considering that these compounds have been well documented to act as redox modulators, and possess higher selectivity and sensitivity in malignant cells [19]. Chen and collaborators [20] reported that the action mechanisms of chemically innovative organic compounds containing selenium in cancer therapy involve mitochondria-mediated pathway induced by ROS, activation of the AMPK pathway, and cell death by necrocytosis, autophagy, mitochondria, or death receptor-mediated pathways. Moreover, the toxicological and chemotherapeutic effects in which organoselenium compounds are involved also include inhibition of thioredoxin reductase [21], DNA damage, apoptosis, and cell cycle arrest [22] [23].

Considering the pharmacological importance of IPs and the biological relevance of organoselenides, molecular hybridization of these two moieties results in certain therapeutic properties of interest [24] [25] [26]. Recently, we reported the synthesis and potential antitumor effect of selenylated IPs using a screening on cancer cell line panel. The compound IP-Se-06 showed promising activity on breast cancer cells [24] and hepatocarcinoma cells [25] with cytotoxicity, inhibition of proliferation, apoptosis, and selectivity of tumor cells at the relatively low micromolar range. Further investigation revealed that IP-Se-06 caused growth inhibition of tumor and inhibited angiogenesis in Ehrlich ascitic carcinoma-bearing mice at 1 mg/kg, the same concentration used for the treatment of animals with doxorubicin (standard antitumor drug for the treatment of hepatocellular carcinoma) [25]. These effects were related to oxidative damage and inhibition of proteins involved in cell proliferation pathways.

In this scientific research article, we report the cytotoxic activity and pharmacokinetic characteristics of IP-Se-06 in the glioblastoma cell line, as well as its molecular mechanism of action through the inhibition of protein kinases involved in tumorigenesis.

## 2. Materials and Methods

### 2.1. Chemicals and Reagents

Dulbecco's Modified Eagle Medium : Nutrient Mixture F-12 (DMEM-F12), fetal bovine serum (FBS), and trypsin were purchased from Gibco (USA). 4′,6-Diamidino-2-phenylindole (DAPI) and tetramethylrhodamine, ethyl ester, perchlorate (TMRE), penicillin, and streptomycin were purchased from Thermo Fisher Scientific Inc. (USA). The following chemicals and reagents were purchased from Sigma-Aldrich: 3-(4,5-dimethylthiazol-2-yl)-2,5-diphenyltetrazolium bromide (MTT), bovine serum albumin (BSA), phosphate-buffered saline (PBS), dimethyl sulfoxide(DMSO), Hanks' balanced salt solution (HBSS), paraformaldehyde, Triton X-100, acridine orange, propidium iodide, 5,5′-dithio-bis(2-nitrobenzoic acid) (DTNB), nicotinamide adenine dinucleotide 2′-phosphate reduced tetrasodium salt hydrate (NADPH), 2′,7′-dichlorofluorescein diacetate (DCFH-DA), *o*-phthalaldehyde (OPT), and MAPK/ERK inhibitor U0126. The following antibodies were used: mouse monoclonal primary antibodies raised against Bcl-xL (Santa Cruz Biotechnology, Inc., sc-8392), p53 (Santa Cruz Biotechnology, Inc., sc-126), Akt (Cell Signaling Technology, USA, #2967), and mTOR (Santa Cruz Biotechnology, Inc., sc-517464); rabbit polyclonal antibodies raised against phospho-Akt (Ser473) (Santa Cruz Biotechnology, Inc., sc-7985), cytocrome *c* (Santa Cruz Biotechnology, Inc., sc-7159), NRF2 (Sigma-Aldrich, Inc., AV39465), *γ*-H2AX (Santa Cruz Biotechnology, Inc., sc-101696), HIF-1*α* (Santa Cruz Biotechnology, Inc., sc-10790), MAP kinase (ERK1/2) (Sigma-Aldrich, Inc., M5670), caspase-1 (Thermo Fisher Scientific Inc., #PA5-87536), p38 MAPK (Cell Signaling Technology, USA, #9212), and phospho-p38 MAPK (Thr180/Tyr182) (Cell Signaling Technology, USA, #9211); and rabbit monoclonal antibody raised against NRLP3 (Cell Signaling Technology, USA, D4D8T). Beta-actin (Santa Cruz Biotechnology, Inc., sc-47778) was used as a loading control. Polyclonal antibodies were purchased from Sigma-Aldrich: goat anti-mouse IgG antibody (Sigma-Aldrich, Inc., AP181P) and polyclonal goat anti-rabbit IgG antibody (Sigma-Aldrich, Inc., AP132P). Chemiluminescence (ECL) detection kit Westar Nova 2.0 was purchased from Cyanagen. All the other chemicals were ACS grade reagents.

### 2.2. Synthesis of Selenylated Imidazo[1,2-a]pyridines

**Figure 1:**
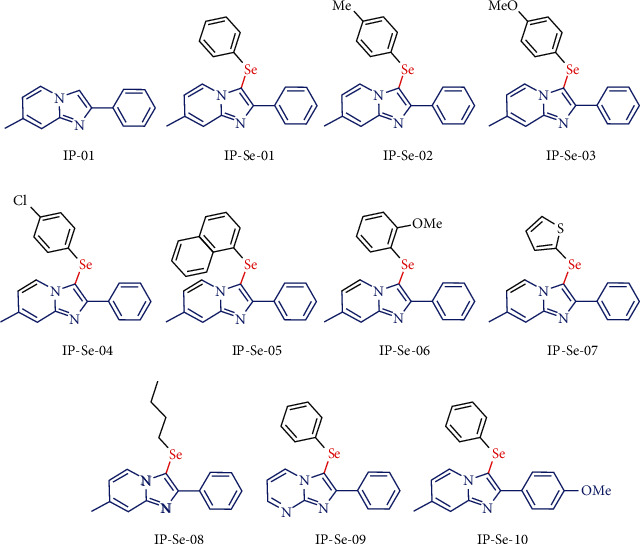
The structures of selenylated imidazo[1,2-*a*]pyridines (IPs).

Selenylated imidazo[1,2-*a*]pyridines (Figure 1) were prepared via direct C(sp^2^)-H bond selenylation of IPs and diorganyl diselenides, as previously described by us [27] [28].

### 2.3. Cell Culture

The cell lines A172 (human glioblastoma) and HT-22 (normal cells from mouse hippocampus) were kindly provided by Dr. Marcelo Farina (Graduate Program in Biochemistry, Center of Biological Sciences, UFSC). The cells were maintained in DMEM-F12 culture medium supplemented with 10% FBS, penicillin (100 U/mL), and streptomycin (100 *μ*g/mL). Cell cultures were seeded into flasks containing medium and maintained in a humidified atmosphere of 95% air and 5% CO_2_ at 37°C.

### 2.4. Cytotoxicity Assay

Cell viability after treatments with selenylated imidazo[1,2-*a*]pyridines (IP-Se-01–IP-Se-10) was analyzed by quantitative colorimetric assay with MTT, according to Mosmann [29]. IP-01, a prototype molecule without the addition of the selenium in its structure, was also tested. A172 and HT-22 cells were cultured in 96-well plates (10^4^ cells/well). After confluence, cells were exposed to selenylated imidazo[1,2-*a*]pyridines (0.1, 1, 10, 100, and 1000 *μ*M) for up to 72 h. After this phase, cell medium was removed and the cells were washed twice with PBS and incubated with MTT solution (0.5 mg/mL) for 2 h. The insoluble formazan was dissolved by adding DMSO (100 *μ*L/well), and colorimetric determination of MTT reduction was measured at 550 nm in a multireader Infinite M200 Tecan. Three independent experiments were conducted, and the results are presented as IC_50_ (half maximal inhibitory concentration).

The selectivity of selenylated imidazo[1,2-*a*]pyridines for glioblastoma cell line was calculated according to as previously reported by Badisa and collaborators [30]: Selectivity Index (SI) = IC_50_ of the compound in normal cell line/IC_50_ of the compound in cancer cell line. According to these authors, a SI value greater than 2 indicates selectivity of the compound to tumor cell.

### 2.5. Biomarkers of Oxidative Stress in A172 Cells

To determine thioredoxin reductase (TrxR) activity and levels of reduced glutathione (GSH), glioblastoma cells were plated (4 × 10^5^/well) into 12-well plates. At confluence, the cells were exposed at low concentration to IP-Se-06 (1 *μ*M) for 6 h. After treatment time, cells were washed twice with PBS and resuspended with PBS/Triton 1%, centrifuged at 5000 g for 5 min and the supernatants were used for the determinations. All results were normalized by the protein content using the Bradford method [31].

#### 2.5.1. Thioredoxin Reductase (TrxR) Activity

TrxR activity was determined in A172 cells according to the method previously described by Arnér and collaborators [32], measuring the NADPH-dependent DTNB reduction to TNB (5-thio-2-nitrobenzoic acid) promoted by TrxR activity. The content of TNB derived from free thiols was measured at 412 nm. The enzyme activity was expressed as *μ*mol·min^−1^·mg protein^−1^.

#### 2.5.2. Reduced Glutathione (GSH) Level

GSH was determined according to Hissin and Hilf [33] using the fluorescent probe *o*-phthalaldehyde, which binds to free -SH groups forming the GS-OPT complex. This complex can be determined by fluorescence excitation at 350 nm and emission at 420 nm. Determinations were expressed in *μ*GSH/*μ*g protein.

### 2.6. Mitochondrial Membrane Potential (*ΔΨ*m) Determination

The loss of mitochondrial transmembrane potential was analyzed using the fluorescent probe TMRE according to as proposed by O'Reilly and collaborators [34], with modifications. A172 cells (4 × 10^5^/well) were plated in 12-well plates. After confluence, the cells were treated with IP-Se-06 (1 *μ*M) for 6 h. After treatment, the cells were washed once with HBSS and incubated with TMRE (1 *μ*M) at 37°C for 20 min. Lastly, the cells were washed with HBSS and the fluorescence intensity was measured at excitation peak of 549 nm and emission of 575 nm.

### 2.7. Chromatin Condensation Detection

DAPI was used to analyze changes in the nuclear morphology of apoptotic cells after DNA damage [35]. A172 cells (0.5 × 10^4^/well) in 24-well plates were treated with IP-Se-06 (1 *μ*M) for 72 h. The cells were then fixed with 4% paraformaldehyde for 10 min at 4°C. The fixed cells were washed three times with PBS (pH 7.4) and stained with DAPI (3 *μ*g/mL) in PBS at 37°C for 10 min. Finally, the cells were washed in PBS, and nuclear morphology was photographed by a fluorescence Olympus IX83 microscope.

### 2.8. Assessment of Cell Death

The cells were seeded (density of 2 × 10^5^/well) into 6-well plates. After confluence, cells were treated with IP-Se-06 (1 *μ*M) for 72 h. After this period, tumor cells were washed with PBS, trypsinized, centrifuged at 1500 g for 5 min, resuspended in PBS, and stained with a solution (6 *μ*L, 1 : 1) of acridine orange (100 *μ*g/mL) plus propidium iodide (100 *μ*g/mL). This staining is used to analyze nuclear changes that characterize apoptotic and necrotic cells according to McGahon and collaborators [36]. Cells (300/glass slide) from triplicates were observed under a fluorescence microscope (Olympus BX41) and categorized as viable, apoptotic, or necrotic.

### 2.9. Cell Cycle Analysis

The PI/RNAse solution from the Immunostep® kit (Salamanca, Spain) was used to determine the DNA content of cells of the different phases of cell cycle by flow cytometry using the protocol proposed by the manufacturer. A172 cells were plated on 6-well plates at a density of 2 × 10^5^/well until confluence and synchronization with nocodazole (30 ng/mL) for 14 h. The cells were treated with IP-Se-06 (1 *μ*M) for 72 h, carefully washed with PBS, and fixed in cold ethanol (70%) overnight at -20°C. Finally, cells were washed again, resuspended in PI/RNAse solution, and incubated for 15 min at room temperature. Cells were analyzed using a BD FACSCanto II (BD Biosciences) flow cytometer. Data were processed using Flowing Software 2.5.1.

### 2.10. Immunocytochemistry Staining for HIF-1*α* Localization

A172 cells were treated with IP-Se-06 (1 *μ*M) for 48 h, fixed with paraformaldehyde (4%) for 15 min and washed three times with PBS. The cells were then treated with blocking solution (0.5% BSA in PBS containing 0.3% of triton X-100) for 1 h at room temperature and incubated with rabbit polyclonal primary antibody HIF-1*α* (Cat. sc-10790, dilution 1 : 500) overnight at 4°C. Following this, the cells were washed three times with PBS and incubated with secondary antibody polyclonal goat anti-rabbit IgG antibody (Cat. AP132P, dilution 1 : 1000) for 1 h. The cell nuclei were stained with DAPI (3 *μ*g/mL). The cell images were acquired from ten randomly chosen fields for each condition using an Olympus IX83 fluorescent microscope.

### 2.11. Clonogenic Assay

To analyze the ability of the organoselenium compound to prevent cell proliferation and the progression of tumorigenesis, the methodology proposed by Franken and collaborators [37] was used. In total, 500 cells per well were seeded in 6-well plates for 24 h. Before treatment with IP-Se-06, two groups were treated with the mitogen-activated protein kinase/ERK kinase inhibitor U0126 (Cell Signaling Technology, USA, Cat. #9903) at 10 *μ*M for 1 h [38]. A172 cells were exposed to IP-Se-06 (1 *μ*M), while untreated cells were maintained as a control. After 72 h of incubation, the cells were washed with PBS, and fresh growth medium DMEM-F12 was added. At 7 days after treatment, colonies were fixed with 10% ethanol, stained with 0.5% crystal violet stain solution, manually counted, and photographed. Finally, cells were discolored with 1 mL of acetic acid 10% for 10 min, and the absorbance was measured at 590 nm in a multireader (Infinite M200 TECAN).

### 2.12. Western Blotting

After treatment with IP-Se-06 (1 *μ*M) for 48 h, A172 cells were harvested with a lysis buffer. The lysates were collected, and the total proteins present in the lysates were quantified according to Lowry [39]. Proteins present in the cell lysates (30 *μ*g) were subjected to SDS-polyacrylamide gel electrophoresis, followed by electroblotting to polyvinylidene fluoride (PVDF) membranes. Nonspecific binding was blocked by incubating the membrane with skimmed milk in TBS buffer containing 0.1% Tween 20 (TBS-T) for 1 h. After primary antibody incubation, membranes were incubated with appropriate HRP-conjugated secondary antibodies for 1 h at room temperature. Immunodetection was performed using an enhanced chemiluminescence (ECL) detection kit. Protein bands were visualized using ChemiDoc MP Imaging System (Bio-Rad) and quantified by densitometry using the freeware ImageJ by Wayne Rasband from the National Institute of Health (USA).

### 2.13. ADMET Profile

The physicochemical properties, pharmacokinetics, druglikeness, and toxicophores of Selenium compound IP-Se-06 were evaluated. Therefore, the spatial data file (.sdf) and canonical SMILES generated with MarvinSketch were submitted to the webserver tools of SwissADME (http://www.swissadme.ch/index.php) [40] [41], ADMETLab (http://admet.scbdd.com) [42], and ADMETLab 2.0 (https://admetmesh.scbdd.com/) [43], respectively.

### 2.14. Statistical Analysis

The results were expressed as the mean ± standard deviation (SD) or as percentages. The data were analyzed using two-way ANOVA followed by the Bonferroni or Tukey-Kramer test. Values of *p* < 0.05 were considered statistically significant. Statistical analysis was obtained from three independent experiments and was performed by GraphPad Prism software, version 8.0 (San Diego, USA).

## 3. Results and Discussion

### 3.1. Cytotoxic Activity of Selenylated Imidazo[1,2-a]pyridines in Glioblastoma Cell

The *in vitro* cytotoxic activity of eleven selenylated imidazo[1,2-*a*]pyridines was measured by the MTT assay. The compound IP-01 without selenium in its chemical structure was used as a prototype molecule for structural modifications. The concentration-dependent effect on A172 and HT-22 cells was examined upon treatment with organoselenium compounds (0.1-1000 *μ*M) following a 72 h procedure. The IC_50_ and SI values are shown in Table 1. Notably, the compound 3-((2-methoxyphenyl)selanyl)-7-methyl-2-phenylimidazol[1,2-*a*]pyridine, named here IP-Se-06, presented the most relevant decrease in glioblastoma cell viability (IC_50_ = 1.8 *μ*M) and also exhibited better selectivity (SI = 36.5) with no significant cytotoxic activity on the mouse hippocampal cell line. Therefore, approximate IC_25_ value of compound IP-Se-06 (1 *μ*M) was used in further experiments in the present study. Notably, compound IP-Se-06 was the most potent compound to induce glioblastoma cell toxicity, a result similar to that obtained by our previous studies with breast cancer cells [24] and hepatocellular carcinoma cells [25]. The low IC_50_ value obtained in A172 cells after treatment with IP-Se-06 (1.8 *μ*M) was of crucial importance, considering that glioblastoma multiforme presents resistance to chemotherapy. TMZ is the common current treatment indicated for glioblastoma multiforme but is related to drug resistance, which is one of the main causes of treatment failure [44]. Previous studies have shown that the concentration of TMZ necessary to inhibit cell viability is at 14.1 *μ*M and 42.6 *μ*M in A172 cells under culture conditions for 144 h (6 days) of drug exposure [5] [45]. Güçlü and collaborators [46] described the novel group of imidazopyridines as a promising candidate for glioblastoma treatment, as these compounds exhibited cytotoxic effect on LN-4055 cells at 10 *μ*M and 75 *μ*M concentrations. Therefore, IP-Se-06 compound showed be a candidate for the development of a novel drug for the treatment of glioblastoma.

### 3.2. IP-Se-06 Changed the Intracellular Redox State of Glioblastoma Cells

**Figure 2:**
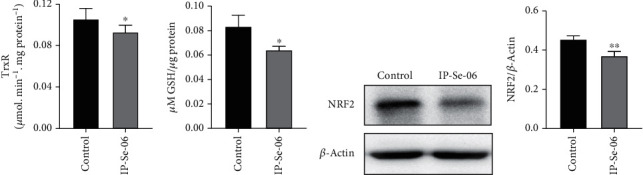
The regulation of redox state by IP-Se-06 in glioblastoma cells. Inhibition of thioredoxin reductase activity (TrxR) (a) and depletion of reduced glutathione (GSH) (b) in A172 cells following exposure for 6 h at IP-Se-06 (1 *μ*M). (c) Immunoelectrophoresis of NRF2 protein levels in total cell lysates. (d) The bar diagrams for NRF2 show relative protein levels from the Western blotting analysis compared to controls; data were normalized to the *β*-actin levels. The data were considered statistically significant at ^∗^*p* < 0.05 and^∗∗^*p* < 0.01 compared to the control group (nontreated cells).

The upregulation of the antioxidant capacity in adaptation to intrinsic oxidative stress in tumor cells can lead to chemoresistance. Nevertheless, altered redox status in malignant cells is a target strategy to improve chemotherapeutic activity and selectivity in the development of new antitumor agents [47]. In this sense, we investigated whether alterations in cell antioxidant defenses were involved with IP-Se-06 cytotoxicity (Figure 2). The data indicated that IP-Se-06 (1 *μ*M) for 6 h significantly decreased TrxR intracellular activity (*p* < 0.05) and levels of GSH (*p* < 0.05), as shown in Figures 2(a) and 2(b), respectively. Similar results for TrxR and GSH depletion after treatment with IP-Se-06 were previously reported by our research group on HepG2 cells (hepatocellular carcinoma) [25]. The levels of NRF2 protein also decreased in response to IP-Se-06 treatment (*p* < 0.01) when compared to controls (Figures 2(c) and 2(d)). Recent evidence has demonstrated that oxidative stress can induce tumor cell death through various signaling pathways [48], thereby opening a new channel for the chemotherapeutic agents. The Trx and GSH systems are required for tumor metabolism and cell survival, and a simultaneously targeting of both systems may be effective in killing tumor cells as a promising strategy for the development of anticancer drug therapy [49]. In this regard, thioredoxin system protein members are overexpressed in various cancers and are related to cell proliferation and aggressive tumor progression, while TrxR inhibition leads to an increase in cellular oxidative stress and consequently induces cell death by apoptosis [50]. He and collaborators [51] described that the redox properties and the anticancer activity of the new organoselenium compounds are associated with the interaction with the active site of thioredoxin reductase.

The GSH system, which maintained the cellular redox balance, utilizes NADPH as an electron donor, like in the Trx system. Also, GSH is highly expressed in tumor tissues, and its activity is relevant in DNA synthesis, regulating mutagenic mechanisms, as well as drug and radiation resistance [52]. A previous study demonstrated that the anticancer activity of selenium compounds occurred via oxidation of reduced glutathione and other thiols, which also revealed apoptogenic effects and alterations in cell cycle progression mediated by ROS [53]. Furthermore, Xie and collaborators [54] showed that a selenium compound could be a strategy for design and further development into effective and low toxicity cancer radiosensitizers, as selenium substitution significantly enhanced X-ray-induced ROS overproduction in cancer cells.

Mutations and activation of transcription factor NF-E2 p45-related factor 2 (NRF2) are common in many cancers and associated with poor prognosis, which are also related to metabolic reprogramming, enhancing the biosynthesis of nucleotides and amino acids, and tumor-promoting inflammation [55]. Pölönen and collaborators [56] described that NRF2 is hyperactivated in glioblastoma patients and contributes to mesenchymal transition, invasion, tumorigenesis, and treatment resistance. Therefore, the potential benefit of NRF2 inhibitors in cancer treatment may trigger a search for agents and provide a novel treatment option against glioblastoma. Accordingly, the modulation of redox status combined with pharmacological NRF2 inhibition promoted by IP-Se-06, as evidenced by our results, can emerge as a promising approach for cancer therapy, including glioblastoma multiforme.

### 3.3. IP-Se-06 Caused Mitochondrial Dysfunction and DNA Damage Leading to Glioblastoma Cell Apoptosis

**Figure 3:**
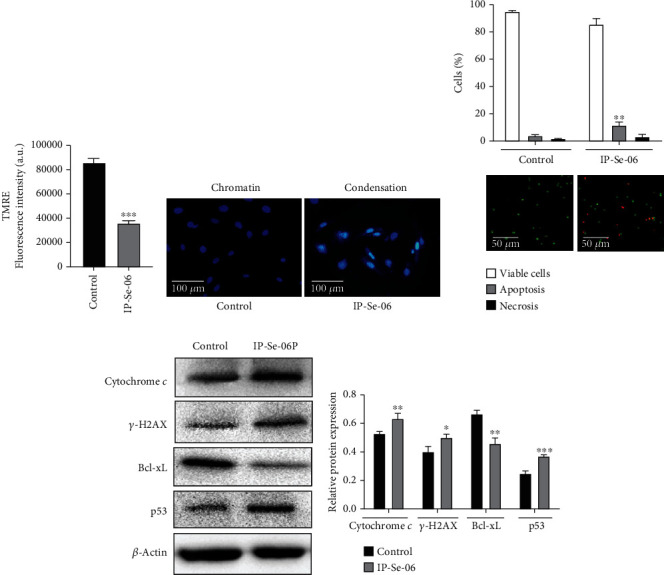
Mitochondrial dysfunction, nuclear damage, and apoptosis induced by IP-Se-06 in glioblastoma cells. (a) Detection of changes in *ΔΨ*m by TMRE fluorescent staining. (b) Cell nucleus morphological changes within chromatin condensation. (c) Detection of apoptotic cells by staining with acridine orange plus propidium iodide. (d) Release of cytochrome *c* into the cytosol, detection of DNA damage biomarker (*γ*-H2AX), Bcl-xL, and p53 levels in A172 cells by Western blotting analysis. The data were normalized to the *β*-actin levels. (e) Densitometric analysis results of cytochrome *c*, *γ*-H2AX and Bcl-xL were quantified with ImageJ. The data were considered as statistically significant at ^∗^*p* < 0.05,  ^∗∗^*p* < 0.01, and^∗∗∗^*p* < 0.001 compared to the control group (nontreated cells).

Intracellular ROS overgeneration changes the intracellular redox state cause massive damage in macromolecules such as DNA, as well as oxidation of proteins and lipids, among other effects, leading to mitochondrial dysfunction. When the mitochondrial membrane potential (*ΔΨ*m) is dissipated, cells enter into an irreversible process of cell death. *ΔΨ*m in A172 cells after treatment with IP-Se-06 (1 *μ*M) for 6 h was analyzed using TMRE fluorescent staining, and the results showed that the fluorescence intensity was reduced (*p* < 0.001) when compared to the control group, indicating the occurrence of depolarization of mitochondrial transmembrane potential (Figure 3(a)). After cells were treated for 72 h morphological changes in the nuclei of the cells were observed using DAPI (Figure 3(b)). The treated cells presented significant morphological changes, with brightly stained and condensed nuclei, observed by the increase in fluorescence, a representative metric for apoptosis, while cells of the control group presented tinted homogeneous nuclei.

Therefore, to investigate whether IP-Se-06 was able to induce apoptosis, A172 cells were treated with IP-Se-06 (1 *μ*M) for 72 h, and cells undergoing apoptosis/necrosis were determined after staining with acridine orange plus propidium iodide. The results demonstrate that the selenylated imidazo[1,2-*a*]pyridine tested in this study was able to induce apoptosis in glioblastoma cells when compared to the nontreated group (*p* < 0.01) (Figure 3(c)). To further investigate the molecular mechanisms underlying compound IP-Se-06 that induce cell death by apoptosis, Western blotting of key apoptotic proteins, including cytochrome *c* in cytosolic fractions, Bcl-xL, p53, and phosphorylated H2AX (*γ*-H2AX), which is a marker of DNA-double strand breaks, was performed (Figures 3(d) and 3(e)). The results demonstrated that levels of cytochrome *c* (*p* < 0.01), *γ*-H2AX (*p* < 0.05) and p53 (*p* < 0.001) were increased in the glioblastoma cell following treatment with IP-Se-06. In addition, the levels of antiapoptotic protein Bcl-xL (*p* < 0.01) decreased in treated cancer cells.

Apoptosis is a programmed cell death process mediated by several signaling pathways triggered by multiple factors including cellular stress and DNA damage. According to Carneiro and El-Deiry [57], the development of agents that directly target mechanisms of apoptosis remains a promising strategy for future clinical practice in oncology. These small molecules can be designed and synthesized to inhibit antiapoptotic Bcl-2 family members, such as Bcl-xL, and these agents are expected to be used in combination with other therapies, such as those targeting oncogenic pathways, immunotherapeutic agents, and radiotherapy.

The p53 protein is a tumor suppressor that activates induction of apoptosis through the Bcl-2-regulated pathway, which is related to cell death by chemotherapeutic drugs that induce DNA damage [58]. GSH depletion can directly modulate mitochondrial membrane potential (MMP) and induces DNA fragmentation, resulting in activation of mitochondrial death cascade [59]. The critical point in the intrinsic pathway of apoptosis signaling is the release of cytochrome *c* from the mitochondria after the collapse of mitochondrial membrane potential, nuclear chromatin condensation, and DNA fragmentation [60].

The release of cytochrome *c* is stimulated by proteins that are encoded by the Bcl-2 family of antiapoptotic and proapoptotic genes [57]. Previous studies by our research group demonstrated that the compound IP-Se-06 was able to induce apoptosis in breast cancer [24] and hepatocarcinoma cells [25]. In the present study, IP-Se-06 disrupted MMP, increased the discharge of cytochrome *c* from mitochondria to cytosol, caused inhibition of the antiapoptotic protein Bcl-xL, caused activation of protein p53, and caused nuclear morphological changes, evidencing that A172 cells were undergoing mitochondria-associated apoptosis.

Furthermore, increasing *γ*-H2AX levels clearly indicated DNA fragmentation after treatment of glioblastoma cells with IP-Se-06. The observations by Aliwaini and collaborators [15] suggest that imidazopyridines may induce apoptosis through p53 regulation. In additions, our results corroborate those of Wang and collaborators [13], showing that novel imidazopyridine derivatives can exert anticancer activity by inducing mitochondrial pathway-mediated apoptosis and indicating such compounds as promising candidates for glioblastoma treatment [46] [16].

### 3.4. IP-Se-06 Induced Cell Cycle Arrest and Inhibition of Akt/mTOR Pathway Proteins

**Figure 4:**
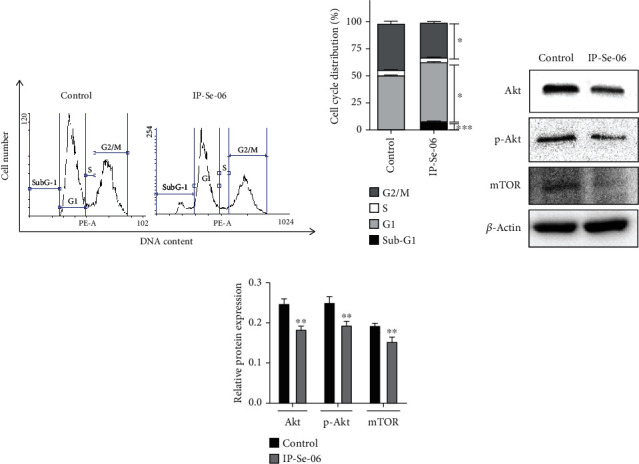
Effects of IP-Se-06 on cell cycle arrest and inhibition of Akt/mTOR pathway. (a) DNA content in cell cycle phases assessed by flow cytometry. (b) Proportion of cells in the cell cycle phases expressed as a percentage of the total number of cells analyzed in the control and treated groups. (c) Levels of proteins Akt, p-Akt, and mTOR after A172 cells were treated with IP-Se-06 (1 *μ*M) at 48 h. Data were normalized to the *β*-actin levels. (d) Densitometric analysis results of Akt, p-Akt, and mTOR were quantified with ImageJ. The data are considered statistically significant at ^∗^*p* < 0.05,  ^∗∗^*p* < 0.01, and^∗∗∗^*p* < 0.001 compared to the control group (nontreated cells).

The effect of compound IP-Se-06 on cell cycle distribution was analyzed by flow cytometry (Figures 4(a) and 4(b)). After 72 h of treatment of A172 cells with IP-Se-06 (1 *μ*M), cells were stained with PI, which is able to enter into the cells and bind to the DNA, allowing evaluation of cell proportions in cell cycle phases based on their DNA levels. The treatment with IP-Se-06 increased cell proportion in sub-G1 peak (*p* < 0.001) compared to the untreated cell group, an indication of cell death by apoptosis [61]. In addition, compound IP-Se-06 caused an increased in the cell population in the G1 phase (*p* < 0.005), while causing a decrease in the G2/M phase cell population (*p* < 0.01). Notably, no significant effect of IP-Se-06 was observed on the S phase of A172 cells. This strongly suggests that IP-Se-06 induce cell cycle arrest by alteration in G1 and G2/M phases in glioblastoma cells. Accordingly, a previous study by our group reported the ability of IP-Se-06 in inhibiting Akt in breast cancer cells [24].

To investigate whether IP-Se-06 causes the same effects in proteins of the Akt pathway of glioblastoma cells, Western blotting for Akt, p-Akt (Akt phosphorylation), and mTOR was performed (Figures 4(c) and 4(d)). The results showed that in A172 cells the treatment with IP-Se-06 (1 *μ*M) after 48 h promoted a significant reduction in levels of Akt (*p* < 0.01), p-Akt (*p* < 0.01), and mTOR (*p* < 0.01), when compared to the control group. According to Koul [62], in glioblastoma cells, Akt is constitutively active due to the loss of protein PTEN (phosphatase and tensin homologue deleted on chromosome ten) function, while activation of Akt is involved in cell survival and proliferation and protects cells from apoptosis.

The constitutive activation of PI3K/Akt pathway led to tumor-promoting effects, as it plays a key role in the G2/M transition [63] and facilitates p53 degradation by increasing MDM2 proto-oncogene expression during malignancy [64]. Also, the phosphorylation of Akt (p-Akt) blocks G2 arrest induced by DNA-damaging agents and radiation [65]. Pharmacological agents that cause deleterious and irreparable damage to DNA integrity drive cells to apoptosis, and this effect can be coupled with ROS formation and suppression of PI3K signaling [66]. Liu and collaborators [67] demonstrated that inhibition of PI3K resulted in the retention of the expression of *γ*-H2AX foci involving double-strand DNA breaks following irradiation, leaving the DNA damage unrepaired and contributing to effectiveness of antitumor treatment. Lee and collaborators [68] showed that inhibition of PI3K/Akt pathway and p38-mitogen-actived protein kinase (MAPK) signaling are involved in DNA-damaging drug-induced apoptosis. Hanahan and Weinberg [69] described that inhibition of Akt in tumor cells has been reported with the induction of downstream mitochondrial apoptotic pathway by alteration in the ratio of Bcl-2/Bax and activation of caspase-3. Thus, the PI3K/Akt/mTOR signaling pathway has been validated as a promising therapeutic strategy by selectively killing or arresting the growth of cancer cells [70].

The ability of imidazo[1,2-*a*]pyridine derivatives to inhibit Akt pathway and cell cycle progression in cancer cells has been recently reported. Aliwaini and collaborators [15] described that imidazo[1,2-*a*]pyridine induces cell cycle arrest and apoptosis, while it is able to inhibit Akt/mTOR pathway in melanoma and cervical cancer cells. Yu and collaborators [14] demonstrated the biological effects of imidazo[1,2-*a*]pyridine derivatives on the PI3K/mTOR dual inhibition, resulting in a significant inhibition of tumor growth in colorectal carcinoma xenografts.

However, although activation of Akt is crucial to glioblastoma cell cycle progression, cell growth, and invasion, the inhibition of Akt and p-Akt is an area that has not yet been extensively explored regarding glioblastoma treatment [71]. Therefore, the inhibition of Akt pathway proteins should be further investigated as therapeutic strategies for glioma. The results from the present study showed that Akt/p-Akt/mTOR expression was downregulated in glioblastoma cells after treatment with IP-Se-06, thus affecting the cell cycle progression. These data also suggest that IP-Se-06 may be considered a promising novel chemotherapeutic drug for glioblastoma treatment.

### 3.5. IP-Se-06 Inhibited HIF-1*α*

**Figure 5:**
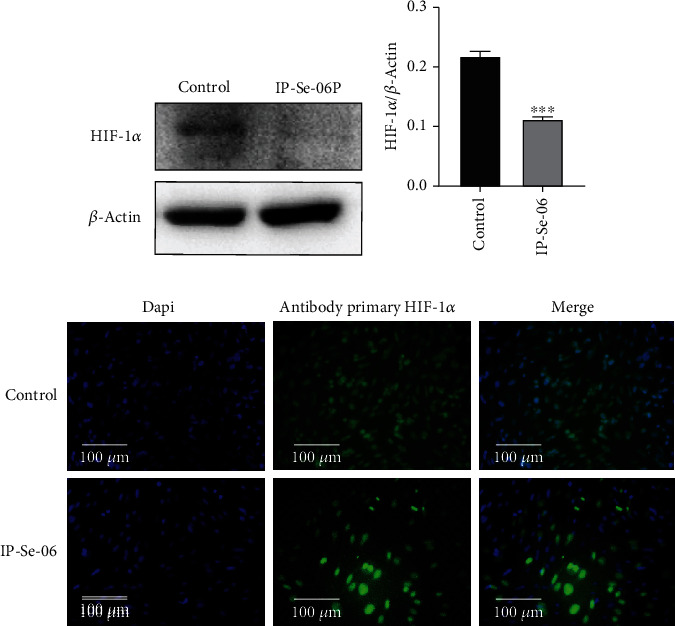
HIF-1*α* after treatment with IP-Se-06. (a) The protein levels of HIF-1*α* detected by Western blot. The data were normalized to the *β*-actin levels. (b) Densitometric analysis result of HIF-1*α* was quantified with ImageJ. (c) HIF-1*α* staining of A172 cells. Cells were stained with anti- HIF-1*α* antibody and DAPI. Scale bar = 100 *μ*m. The data were considered as statistically significant at ^∗∗∗^*p* < 0.001 compared to the control group (nontreated cells).

Several studies have shown the relationship between the PI3K/Akt/mTOR pathway and the hypoxia-inducible factor-1*α* (HIF-1*α*), in the migration, invasion, and drug-resistant phenotype of human glioblastoma under hypoxia [6] [72]. Considering the importance of HIF-1*α* and its relationship with Akt signaling, we examined the effect of IP-Se-06 treatment in the levels of HIF-1*α* in glioblastoma cells. As show in Figures 5(a) and 5(b), the HIF-1*α* levels were significantly decreased (*p* < 0.01) after treatment with IP-Se-06 (1 *μ*M) for 48 h. An immunocytochemistry assay was carried out to investigate the location of HIF-1*α* protein within the cells following IP-Se-06 treatment (Figure 5(c)). HIF-1*α* was detectable in untreated cells at much higher levels than in IP-Se-06-treated cells, while this protein seemed not to be translocated to the nucleus in the treated group, indicating HIF-1*α* pathway inhibition in response to IP-Se-06 exposure.

In a tumor microenvironment under hypoxic conditions, inflammatory cytokines, growth factors, and signaling molecules stimulate the activation of the PI3K/Akt pathway, while the stimulation of mTOR increases the synthesis rate of proteins, including HIF-1*α* [73]. Furthermore, HIF-1*α* is a cytoplasmatic transcription factor that, after its activation, shows an increase in cellular levels and translocate to the nucleus of cell, where it dimerizes with HIF-1*β* to provide the active transcription factor HIF-1. Subsequently, HIF-1*α* plays an essential role during metastasis and angiogenesis in solid tumors [74]. Previous findings have shown that organoselenium compounds inhibit HIF-1*α*, leading to strong anticancer effects on human cancer cell lines [23] [75].

Moreover, the anticancer capacity of novel imidazopyridine analogues has been related with a decreased expression of HIF-1*α* [76]. Accordingly, in the present study, IP-Se-06 significantly inhibited HIF-1*α* levels and promoted translocation of the nucleus in A172 cells, indicating that it is targeting HIF-1*α* through the Akt/mTOR pathway.

### 3.6. Antiproliferative Activity of IP-Se-06 on A172 Cell by ERK 1/2 Inhibition

**Figure 6:**
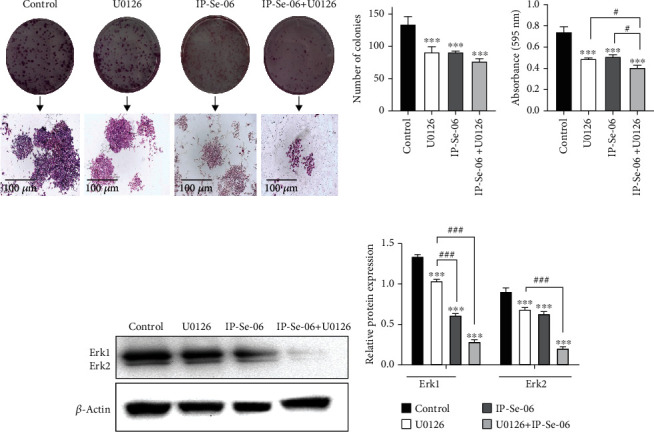
Clonogenic assay and ERK 1/2 protein levels after IP-Se-06 exposure on glioblastoma cells. (a) Crystal violet-stained cultures, scale bar = 100 *μ*m, (b) number of colonies formed, (c) colonies discolored with acetic acid 10% and the absorbance measured at 590 nm after treatment with IP-Se-06 (1 *μ*M) at 72 h and/or MEK/ERK inhibitor (U0126) at 10 *μ*M for 1 h. (d) Western blotting of ERK 1/2 in A172 cells treated with IP-Se-06 (1 *μ*M) at 48 h and MEK/ERK inhibitor (U0126) at 10 *μ*M for 1 h. *β*-Actin was used as loading control. (e) Densitometric analysis result of ERK 1/2 was quantified with ImageJ. The data were considered as statistically significant at ^∗∗∗^*p* < 0.001 compared to the control group (nontreated cells). ^#^*p* < 0.05 and ^###^*p* < 0.001 indicate a significant difference compared to the treatments.

Extracellular signal-regulated kinase 1/2 (ERK 1/2) signaling is required for glioblastoma cell proliferation, invasion, and tumorigenesis [77], and ERK activity is also associated with resistance to the treatment of glioblastoma [8]. Therefore, IP-Se-06 treatment was evaluated to identify its potential to inhibit ERK 1/2 protein involved in proliferation in glioblastoma cell line. Clonogenic survival was determined in A172 cells after treatment with IP-Se-06 (1 *μ*M) for 72 h and a potent MEK/ERK inhibitor (U0126) for 1 h (Figures 6(a)–6(c)). The antiproliferative effect of IP-Se-06 was evident in glioblastoma cells, as the treatment decreased the rate of colony formation (*p* < 0.001) when compared to nontreated cells (Figures 6(a) and 6(b)). The reduction of clonogenicity and synergistic effect for the combination of IP-SE-06 and U0126 was demonstrated after the cells were decolorized with acetic acid (Figure 6(c)). The number of cells in the colonies decreased after treatment with IP-Se-06 (*p* < 0.01) compared to controls, which was even lower when the treatment was carried out concomitantly with U0126 (*p* < 0.05) when compared to the treatments alone.

After showing the role of IP-Se-06 in downregulation the proliferation of glioblastoma cells, this result suggests the inhibition of the commonly aberrant active pathway ERK 1/2; the levels of this protein were analyzed by the immunoblot assay (Figures 6(d) and 6(e)). The present data provide an important reduction of ERK 1/2 levels in A172 cell by IP-Se-06 compared with controls (*p* < 0.001). The combination of IP-Se-06 and the MEK/ERK inhibitor (U0126) resulted in synergistic inhibition of the molecular levels of ERK 1/2 when compared to nontreated cells (*p* < 0.001), as well as when the cells were treated with U0126 alone (*p* < 0.001). These findings strongly suggest the interesting notion that reduction in ERK 1/2 levels by IP-Se-06 treatment might be involved in inhibition of human glioblastoma proliferation in the cell line. Our results agree with those of Bu and collaborators [78], who reported that the imidazopyridine derivatives showed an antiproliferative potency against diffuse large B-cell lymphoma cells by the inhibition of mitogen-activated protein kinase (MAPK). In this regard, Li and collaborators [79] demonstrated that the molecular mechanism of anticancer activity of imidazopyridines involves a significant suppression of the oncogenic MEK/ERK kinase phosphorylation in human prostate cancer cells. In summary, imidazopyridines with ERK inhibiting effect may have a potential therapeutic benefit for cancer treatment, and our study is the first to show ERK 1/2 inhibition by a selenylated imidazo[1,2-*a*]pyridine derivative in human glioblastoma cells.

### 3.7. IP-Se-06 Causes Inhibition of MAPK and Inflammasome Proteins

**Figure 7:**
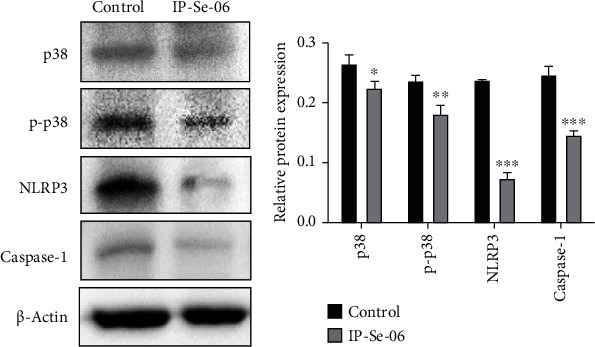
Treatment with IP-Se-06 (1 *μ*M) at 48 h downregulated p38 mitogen-activated protein kinase and inflammasome complex protein in A172 glioblastoma cells. (a) Immunoblotting analysis detected the downregulation of p38, p-p38, NLRP3 and caspase-1 protein levels. *β*-Actin was used as a loading control. (b) Densitometric analysis results of p38, p-p38, NLRP3 and caspase-1 were quantified with ImageJ. The data were considered as statistically significant at ^∗^*p* < 0.05,  ^∗∗^*p* < 0.01, and^∗∗∗^*p* < 0.001 compared to the control group (nontreated cells).

It is known that intracellular oxidative stress can modulate redox-dependent Akt and mitogen-activated protein kinase (MAPK) signal transduction pathways, which can lead to apoptosis of cancer cells [80]. The p38 mitogen-activated protein kinase is a member of MAPK family proteins and supports a key role in mediating resistance to anticancer treatments, also promoting cell growth, survival signaling, proliferation, migration, and inflammation [81]. Therefore, we evaluated whether IP-Se-06 could induce changes in p38 and p-p38 protein levels as well as in inflammasome complex proteins. For this purpose, A172 cells were exposed to IP-Se-06 (1 *μ*M) for 48 h and the proteins were analyzed by immunoblotting (Figures 7(a) and 7(b)). The results showed that IP-Se-06 significantly decreased the levels of p38 (*p* < 0.05) and also the phosphorylation of this protein (*p* < 0.01). Subsequently, the same treatment demonstrated that IP-Se-06 also decreased the levels of inflammasome complex proteins, nucleotide-binding oligomerization domain-like receptor (NLRP3) (*p* < 0.001), and caspase-1 (*p* < 0.001).

These findings clearly indicate that IP-Se-06 induces the inhibition of MAPK family proteins and can regulate the inflammation process in A172 glioblastoma cells. According to Rodríguez-García and collaborators [7], activation of the p38 MAPK and PI3K/Akt signaling pathways converge with activation of Smad signaling and these mechanisms mediate the reprogramming of glioblastoma cells. Furthermore, p38 seems to play a predominant role in NLRP3 inflammasome activation, in which adenosine triphosphate (ATP) drives the activation of caspase-1, upregulating the proinflammatory profile of different diseases [82]. Although NLRP3 activation has been reported to be associated with tumorigenesis, its expression and function in human glioblastoma remain unclear. Yin and collaborators [83] reported that NLRP3 in human glioblastoma regulates cellular proliferation and metastasis via epithelial-mesenchymal transition and PTEN/Akt signaling pathway, while Souza and collaborators [84] described that an organoselenium compound protects against the inflammatory response in the head kidney and spleen of grass carp via downregulation of NLRP3 inflammasome.

Nevertheless, so far, there are no reports in the scientific literature regarding the inhibition of the inflammasome complex by organoselenium compounds or imidazopyridines derivatives for the treatment of cancer. The present data strongly suggest that the inhibition of p38 protein and its phosphorylation by IP-Se-06 downregulated NLRP3 and caspase-1, revealing a promising pathway that may offer novel insights for the development of new glioblastoma clinical therapeutic strategies.

### 3.8. ADMET Profile of IP-Se-06

**Figure 8:**
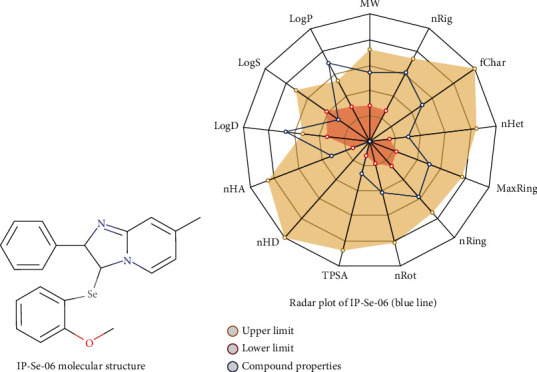
Chemical structure of 3-((2-methoxyphenyl)selanyl)-7-methyl-2-phenylimidazol[1,2-*a*]pyridine, named IP-Se-06 (a). Radar plot showing some physicochemical and pharmacokinetic properties of IP-Se-06 (b).

Estimation of pharmacokinetic properties plays a central role in the early phases of new drug discovery. *In silico* models to predict ADMET profile (Adsorption, Distribution, Metabolism, Excretion, and Toxicity) of compounds have been incorporated into drug development to avoid failures due to poor information regarding pharmacokinetics and toxicity [85]. Some of the main pharmacokinetic characteristics of compound IP-Se-06 are shown in Table 2.  Figure 8(a) shows the structure of IP-Se-06, while Figure 8(b) shows the radar map depicting the physicochemical space of IP-Se-06. The parameter LogS correlated to the compound aqueous solubility was lower than optimal values (0.5 log mol/L). In addition, its solubility can be influenced by the number of sp^3^ hybridized carbons/total carbon count when it is lower than the suitable value (Table 2).

Additionally, IP-Se-06 unsaturation was slightly higher than the ideal value (0.25 < Fraction Carbon sp3 < 1) and reflected its molecular tetrahedral geometry correlated with hybridization Csp3 (Figure 8(b)). Also, IP-Se-06 has higher LogP values associated with low topological polar surface area (TPSA) (26.53 angstroms) (Table 2). Therefore, the pharmacokinetic properties linked with absorption and distribution of this compound suggest its potential to penetrate plasma membranes as well as cross the blood-brain barrier and be absorbed through the intestinal epithelium (Table 2).

Interestingly, IP-Se-06 satisfies the Golden Triangle Rule (200 ≤ MW ≤ 50; -2 ≤ LogD ≤ 5), and for this reason, it likely possesses a favorable ADMET profile. Thus, in general, ADMET evaluation pointed out its adequacy to the druglikeness rules, when considering the main rules (Lipinski; Veber; and others). However, IP-Se-06 showed a potential of mutagenicity and hepatotoxicity which were predicted in silico according to Ames mutagenicity (category 1; probability 0.754) and Drug-Induced Liver Injury (DILI) (category 1; probability 0.788) (Table 2). Among all toxiphore rules, IP-Se-06 has only one caveat related to its N-heteroatom ring merged with another aromatic ring (Table 2). These toxiphore rules include acute toxicity, genotoxic and carcinogenicity and nongenotoxic features inside compound structures. This probably could be related to its moderate clearance (9.819 mL/min/kg) despite its half-life (*T*_1/2_ = 0.27 h), which was calculated with ADMETLab 2.0. Table 2 showed that IP-Se-06 was considered as a nonsensitizer for skin and does not present any PAINS structural alert (Category 0). Thus, further structural developments will be necessary to improve this compound's pharmacokinetics and druglikeness with low toxicity. In this regard, our previous *in vivo* study showed antitumor effects under the lowest concentrations of IP-Se-06 [25].

## 4. Conclusions

The present study demonstrated that IP-Se-06 presented good pharmacological properties (absorption, distribution, metabolism, excretion, and toxicity). IP-Se-06 at low concentration (1 *μ*M) caused changes in intracellular redox status in malignant cells by inhibition of TrxR activity and levels of GSH and NRF2 proteins. The oxidative stress caused chromatin condensation and DNA damage in A172 glioblastoma cells, and these effects stimulated intrinsic apoptosis by disrupting the mitochondrial membrane integrity while promoting the release of cytochrome *c* into cytosol, as well as the decrease of Bcl-xL levels concomitant to increased p53 levels. The data also suggests that cell cycle arrest and apoptosis induced by IP-Se-06 may be mediated by Akt/mTOR/HIF-1*α* pathway inhibition. Furthermore, IP-Se-06 proved to be effective in downregulation of members of MAPK family proteins. In addition, the inhibition of MEK/ERK 1/2 signaling by IP-Se-06 resulted in antiproliferative activity, as well as in the inhibition of p38 mitogen-activated protein kinase, thereby leading to inhibition of inflammasome complex proteins, such as NLRP3 and caspase-1 in glioblastoma cells. Altogether, these results suggest that IP-Se-06 could be a potential candidate for further development of a most promising drug for glioblastoma treatment.

## Figures and Tables

**Table 1:** IC_50_ values and selective index (SI) for treatment of glioblastoma cells (A172) and normal cell from mouse hippocampal (HT-22) cells with selenylated imidazo[1,2-*a*]pyridine for 72 h.

	IC_50_ (*μ*M)		
Compounds	A172	HT-22	Selectivity Index (SI)
IP-01	76.3	153.4	2.0
IP-Se-01	98.8	75.4	0.7
IP-Se-02	73.2	143.5	1.9
IP-Se-03	27.6	3.9	0.1
IP-Se-04	43.6	305.7	7.0
IP-Se-05	20.8	206.2	9.9
IP-Se-06	1.8	65.0	36.5
IP-Se-07	27.9	10.7	0.4
IP-Se-08	20.9	31.2	1.5
IP-Se-09	21.8	6.0	0.3
IP-Se-10	18.8	30.0	1.6

*SI* = *IC*_50_ of the compound in normal cell line/IC_50_ of the compound in the A172 cell line. Data from three independent experiments. IC_50_: half-maximal inhibitory concentration; SI: Selectivity Index.

**Table 2:** *In silico* ADMET (Absorption, Distribution, Metabolism, Excretion and Toxicity) analysis for compound IP-Se-06.

Property		Lipophilicity LogP			Pharmacokinetics [probability]^∗^	Water solubility LogS			
Molecular weight (g/mol)	393.34 394.06^∗∗^	iLOGP	0.0	GI absorption2	High Cat. 1^∗^ [0.813]	CYP2C9 substrate	No. Cat. 0^∗^ [0.493]^∗^	ESOL method	-6.05 (Poorly soluble)
Heavy atoms	25	XLOGP3	5.42	BBB permeant3	Yes Cat.1^∗^ [0.917]	CYP2C19 inhibitor	Yes Cat. 1^∗^ [0.837]^∗^	Ali method	-5.73 (Moderately soluble)
Aromatic heavy atoms	21	WLOGP	2.97	P-gp4 inhibitor^∗^	Yes Cat. 1 [0.701]^∗^	CYP2C19 substrate	Yes Cat. 1^∗^ [0.579]^∗^	LogS (SILICOS–IT)	-7.83 (Poorly soluble)
Rotatable bonds	4	MLOGP	3.37	P-gp4 Nonsubstrate^∗^ (N + O ≤ 4)	Yes Cat. 0^∗^ [0.108]^∗^ (N + O = 3)	CYP2D6 inhibitor	Yes Cat. 1^∗^ [0.536]^∗^	LogS	-5.723^∗∗^
H-bond acceptor	2 (3)	SILICOS-IT	3.52	CYP1A2 inhibitor	No Cat.1^∗^ [0.848]^∗^	CYP2D6 substrate	Yes Cat. 1^∗^ [0.556]^∗^		
H-bond donor	0	Consensus	3.06	CYP1A2 substrate	Yes Cat.1^∗^ [0.575]^∗^	CYP3A4 inhibitor	Yes Cat. 0^∗^ [0.475]^∗^		
LogP				5.07^∗∗^					
TPSA**1** (Å^2^)	26.53	Wildman-Crippen LogP^∗^	2.97 (Optimal)	CYP2C9 inhibitor	Yes Cat.1^∗^ [0.683]^∗^	CYP3A4 substrate	Yes Cat. 1^∗^ [0.508]^∗^		
Druglikeness rules			Medicinal chemistry				Toxicity (%)		
Lipinski	Yes	Egan	Yes	PAINS5 alerts	0	Leadlikeness	2	AMES6	Cat. 1^∗^ [0.754]^∗^
Ghose	Yes	Muegge	1	Brenk structural alert	1	Synthetic accessibility score	3.51 2.733^∗∗^	SkinSen7	Cat. 0^∗^ [0.183]^∗^
Veber	Yes	Bioavailability score	0.55	Drug-likeness (Desirability)	0.499^∗∗^ (Unattractive)			DILI8	Cat. 1^∗^ [0.788]^∗^
Golden triangle	Accepted	Compounds satisfying the Golden triangle rule May have a more favorable ADMET profile						FDAMDD9	Cat. 0^∗^ [0.468]^∗^

^1^Topological polar surface area; ^2^gastrointestinal absorption; ^3^blood-brain barrier; ^4^P-glicoprotein; ^5^pan assay interference structures; ^6^mutagenicity; ^7^skin permeation; ^8^drug-induced liver injury; ^9^maximum recommended daily dose; ^∗^ADMETLab; ^∗∗^ADMETlab 2.0.

## Data Availability

The results used to support the findings of this study are available from the corresponding author upon request.
